# PLS3 Is a Prognostic Biomarker and Correlates with Immune Infiltrates in Head and Neck Squamous Cell Carcinoma

**DOI:** 10.3390/cancers17233882

**Published:** 2025-12-04

**Authors:** Yuhong Wang, Kunyi Chen, Guoli Tian, Chen Hou, Yingzhao Huang, Fan Song, Ming Zhang, Jinsong Hou

**Affiliations:** 1 Hospital of Stomatology, Sun Yat-Sen University, Guangzhou 510055, China; 2 Guanghua School of Stomatology, Sun Yat-Sen University, Guangzhou 510055, China; 3 Guangdong Provincial Key Laboratory of Stomatology, Sun Yat-Sen University, Guangzhou 510080, China

**Keywords:** immune regulation, CD8^+^ T cells, epithelial–mesenchymal transition, head and neck squamous cell carcinoma, PLS3

## Abstract

Background: Head and neck squamous cell carcinoma (HNSCC) features aggressive progression and a complex immunosuppressive microenvironment, contributing to poor patient prognosis and highlighting the need to identify key molecular drivers. The actin‑binding protein PLS3 (Plastin‑3) is involved in tumor metastasis, yet its expression, function, and therapeutic potential in HNSCC remain unclear. Methods: PLS3 expression was analyzed via polymerase chain reaction (PCR), Western blot (WB), and immunohistochemistry (IHC). PLS3 was knocked down in vitro to assess its effects on HNSCC cell functions, and in vivo models were established to evaluate tumor progression and immune microenvironment. Bioinformatics analyses explored the relationship between PLS3 and tumor immunity. Results: PLS3 was upregulated in HNSCC tissues and correlated with adverse clinical outcomes. Knockdown of PLS3 inhibited cell proliferation, migration, and invasion in vitro, and suppressed tumor growth in vivo. Mechanistically, PLS3 promoted HNSCC progression by activating the epithelial-mesenchymal transition (EMT) program. Bioinformatics and animal studies further linked PLS3 overexpression to an immunosuppressive microenvironment characterized by reduced CD8+ T-cell infiltration and downregulated chemokine expression. Conclusions: This study elucidates the oncogenic role of PLS3 in HNSCC and supports its potential as a prognostic biomarker and therapeutic target.

## Simple Summary

Head and neck cancer is a severe disease that often spreads aggressively and is difficult to treat, partly because the environment around the tumor can weaken the body’s immune defenses. This highlights the need to find key molecules that drive the cancer’s growth. In this study, we investigated a protein called PLS3, which is known to help cells move and spread. We found that PLS3 is present at high levels in head and neck cancer tissues, and patients with more PLS3 tend to have worse outcomes. When we reduced PLS3 in cancer cells, they grew more slowly, moved less, and were less invasive. In animal tests, lowering PLS3 also slowed tumor growth. We discovered that PLS3 works by helping cancer cells become more mobile and invasive. Additionally, high PLS3 levels were linked to a weaker immune response against the tumor. This research is the first to show the important role of PLS3 in driving head and neck cancer progression. It suggests that measuring PLS3 could help predict patient survival, and that targeting this protein may offer a new strategy for future treatments.

## 1. Introduction

Head and neck squamous cell carcinoma (HNSCC), from the mucosal epithelium of the oral cavity, oropharynx, and larynx, is the most prevalent malignancy in the head as well as neck region. According to 2022 GLOBOCAN statistics, its annual new cases have surpassed 930,000, with 5-year survival rates persistently stagnant at 40–60% due to early lymph node metastasis and aggressive biological behavior [1,2]. Epidemiological projections indicate that driven by smoking, alcohol consumption, and HPV infection, the annual incidence of HNSCC is predicted to surge by 30% by 2030, reaching approximately 1.21 million cases worldwide, underscoring its escalating disease burden [3]. Current clinical management, including surgery combined with chemoradiotherapy, effectively controls localized tumors; however, approximately 60% of advanced patients experience treatment failure due to local recurrence, distant metastasis, or acquired immune resistance [4,5]. While immune checkpoint inhibitors targeting PD-1/PD-L1 have recently offered hope for advanced HNSCC, their objective response rate (ORR) remains limited to 15–20% [6], and existing biomarkers fail to reliably stratify beneficiaries due to spatiotemporal heterogeneity and suboptimal predictive efficacy [7]. This therapeutic impasse highlights that HNSCC progression relies not only on tumor cell metastatic potential but also on dynamic remodeling of an immunosuppressive microenvironment to evade host immune surveillance [8]. Consequently, identifying pivotal molecules that coordinately regulate metastatic phenotypes and immune evasion represents a critical step toward overcoming current therapeutic limitations.

The composition and functional dysregulation of the tumor microenvironment (TME) are central pathological features driving immune evasion in HNSCC. Single-cell transcriptomic studies reveal that the immune microenvironment of primary HNSCC tumors and metastases exhibits high similarity, yet metastatic lymph nodes contain a larger proportion of exhausted T cells, which is linked to intensified local immunosuppression [9]. This underscores the important role of TME alterations in HNSCC malignant progression. Notably, epithelial–mesenchymal transition (EMT), a critical promoter of cancer cell invasion as well as metastasis, forms a bidirectional regulatory network with TME through multifaceted mechanisms: on one hand, EMT-associated transcription factors (e.g., ZEB1) impair CD8^+^ T cell infiltration as well as diminish anti-PD-1 therapeutic efficacy [10], while core EMT regulators (Snail, Twist) activate the TGF-β/Smad pathway to induce T cell exhaustion [11] as well as recruit myeloid-derived suppressor cells [12]. Mesenchymal-phenotype tumor cells downregulate MHC-I expression [13], attenuating antigen presentation and promoting regulatory T cell (Treg) differentiation. This vicious cycle of EMT–immune crosstalk may be orchestrated by molecular hubs (e.g., cytoskeletal regulators) that integrate cross-mechanistic signaling [14]. However, the precise regulatory networks and critical nodal molecules remain elusive.

Plastin-3 (PLS3), an actin-bundling protein critical for cytoskeletal remodeling, has been implicated in tumor metastatic cascades through its regulation of mechanosensory signaling [15]. Accumulating evidence highlights its pro-metastatic roles across malignancies: In the context of colorectal cancer (CRC), PLS3 suggests heightened expression in circulating tumor cells (CTCs) of individuals who develop metastasis, correlating with poor prognosis [16]. PLS3 copy number gain drives EMT via TGF-β signaling, conferring invasive capabilities to CRC cells [17]. Similarly, elevated PLS3 in CTCs serves as a biomarker for recurrence risk and unfavorable outcomes in breast cancer [18]. Within pancreatic cancer, PLS3 facilitates tumor cell proliferation through activating the PI3K/AKT pathway [19]. In HNSCC, PLS3 knockdown attenuates LONP2-driven oncogenicity, further supporting its tumor-promoting function [20]. Notably, single-cell transcriptomic profiling of T follicular helper cell lymphomas identified PLS3 as a tumor-specific marker linked to immune evasion and drug resistance [21]. Despite its established role in tumor progression, PLS3’s contribution to immune microenvironment regulation remains largely unexplored. We thus propose a novel hypothesis: PLS3 may drive HNSCC progression by synergistically activating EMT and remodeling an immunosuppressive TIME, thereby establishing a bidirectional “pro-metastatic–immune evasive” axis.

This study systematically deciphered the dual mechanisms of PLS3 in HNSCC by integrating public database analyses and experimental validation. First, TCGA-based analysis revealed high PLS3 expression was remarkably related with poor prognosis in HNSCC patients. Immunohistochemistry (IHC) further confirmed elevated PLS3 levels within tumor tissues as opposed to normal control samples. Functional assays in vitro and in vivo illustrated that PLS3 knockdown suppressed tumor growth, migration, and metastatic processes. Mechanistically, PLS3 was shown to induce EMT through a previously undefined axis. Bioinformatics analyses revealed that high PLS3 expression correlated with suppressed secretion of T cell-recruiting chemokines (e.g., CCL19, CXCL1) and a marked reduction in CD8^+^ T cell infiltration (*p* < 0.01), suggesting that PLS3 impairs antitumor immunity by disrupting chemokine-mediated immune cell recruitment. These findings unveil PLS3 as a molecular bridge linking EMT activation to immunosuppressive microenvironment remodeling in HNSCC, providing novel insights into therapeutic strategies targeting tumor cell–immune microenvironment crosstalk.

## 2. Materials and Methods

### 2.1. Culture

The Guangdong Provincial Key Laboratory of Oral Medicine (Guangzhou, China) provided the HNSCC cell lines HSC3 and SCC25, which were cultivated in DMEM medium (Gibco, Grand Island, NY, USA) with 10% fetal bovine serum (FBS; Gibco, USA) added. The incubation process was carried out at 37 °C in a humidified environment with 5% CO_2_.

### 2.2. Quantitative Real-Time PCR (RT-qPCR)

Total RNA isolation adopted the RNA Rapid Purification Kit (ES Science, Shanghai, China; Cat. RN001), as well as RNA concentration measurement utilized NanoDrop spectrophotometry. cDNA synthesis applied the HiScript II One-Step RT-PCR Kit (Vazyme, Nanjing, China; Cat. P611-01) following the manufacturer’s instructions. RT-qPCR was carried out with SYBR Green Premix on a StepOnePlus Real-Time PCR System (Applied Biosystems, Foster City, CA, USA), with each sample analyzed in triplicate. Relative expression levels were calculated using the ΔCt method and normalized to *ACTB* (β-actin). Primer sequences for *PLS3* as well as *ACTB* were as follows:

*PLS3*: 

Forward: 5′-TGGAGGGCAAGACCTGAATG-3′

Reverse: 5′-CACAACTGCCAAACTGGAGC-3′

*ACTB*:

Forward: 5′-CTCGCCTTTGCCGATCC-3′

Reverse: 5′-ATCCTTCTGACCCATGCCC-3′

### 2.3. Western Blotting

HNSCC cell lines were lysed using RIPA lysis buffer (CWBIO, Beijing, China; Cat. CW2333S), and supernatants were harvested following centrifugation of lysates. Protein quantification was conducted via a BCA assay. Equal protein quantities were separated by 10% SDS-PAGE gels (CWBIO; Cat. P0014B) as well as electrotransferred to polyvinylidene difluoride membranes (Millipore, Billerica, MA, USA; Cat. ISEQ00010).

Membranes were blocked with 5% skim milk (Corning, Corning, NY, USA; Cat. 232100) in Tris-buffered saline with 0.1% Tween-20 (TBST) for 1 h at room temperature. Overnight incubation at 4 °C followed with the following primary antibodies: anti-PLS3 (Proteintech, Rosemont, IL, USA, cat. no. 12917-1-AP, 1:1000 dilution), anti-GAPDH (Affinity Biosciences, Cincinnati, OH, USA, cat. AF7021, 1:10,000), anti-E-cadherin (Proteintech, cat. 20874-1-AP, 1:20,000), anti-N-cadherin (Cell Signaling Technology, Danvers, MA, USA, cat. 13116, 1:1000), and anti-Vimentin (Proteintech, cat. 60330-1-Ig, 1:20,000).

The next day, horseradish peroxidase-conjugated secondary antibodies were leveraged for 1 h at room temperature. After TBST washing, protein bands were visualized via Immobilon Western chemiluminescent HRP substrate (Millipore; Cat. WBKLS0500) as well as imaged with a ChemiDoc XRS+ system (Bio-Rad, Hercules, CA, USA).

### 2.4. Immunohistochemistry (IHC)

HNSCC tumor specimens as well as non-tumorous adjacent tissues were procured at Sun Yat-sen University Hospital of Stomatology (Guangzhou, China). Written informed consent was attained from all participants prior to inclusion, with ethical compliance ensured under the Helsinki Declaration principles. The research protocol was approved by the Ethics Committee of Sun Yat-sen University Hospital of Stomatology (Approval Code: KQEC-2022-65-01).

Tissue specimens were fixed in 10% formalin as well as embedded in paraffin, followed by preparing 4-μm-thick serial sections that were baked on glass slides at 60 °C for 2 h. Deparaffinization with xylene as well as rehydration through graded ethanol were sequentially performed, succeeded by antigen retrieval using EDTA-citrate buffer (pH 9.0). Endogenous peroxidase activity was quenched with hydrogen peroxide. Sections were blocked with 10% goat serum (Boster Biological Technology, Wuhan, China; Cat. AR0009) for 30 min at room temperature to block nonspecific binding, then incubated overnight at 4 °C with primary anti-PLS3 antibody (Proteintech, cat. 12917-1-AP, 1:400 dilution). The next day, HRP-conjugated polymer secondary antibody (GeneTech, San Francisco, CA, USA, Cat. GK600710) was leveraged at 37 °C for 30 min. After hematoxylin counterstaining, slides were dehydrated in graded ethanol and mounted with neutral resin (Servicebio, Wuhan, China; Cat. WG10004160). Images were finally captured using a Leica Aperio AT2 digital slide scanner (Leica Microsystems, Wetzlar, Germany). IHC scoring was performed based on the H-score method, calculated as:

(1) H-score = (Percentage of positive cells × Staining intensity)

where percentage of positive cells was classified into 0 (0%), 1 (1–25%), 2 (26–50%), 3 (51–75%), or 4 (76–100%), and staining intensity was scored as 0 (negative), 1 (weak), 2 (moderate), or 3 (strong). Final H-scores ranged from 0 to 300.

### 2.5. siRNA Transfection

Three small interfering RNAs (siRNAs) designed to target PLS3 were applied for knocking down its expression in HSC3 and SCC25 cell lines:

siPLS3#1: 5′-GGUUGCAGACAGUUUGUUATT-3′

siPLS3#2: 5′-GUAGACAUGUUAUACCAAUTT-3′

siPLS3#3: 5′-CUGCUUAGAUGGGCAAACUTT-3′

A scrambled siRNA (5′-UUCUCCGAACGUGUCACGUTT-3′), displaying no sequence similarity to any known human genes, functioned as a negative control (NC). siRNA oligos were diluted in serum-free Opti-MEM I medium. For transfection complex formation, diluted siRNA oligos were mixed with PepMute™ Transfection Reagent (SignaGen, Frederick, MD, USA; Cat. SL100566) at a ratio customized for HNSCC cells. The mixture incubated at room temperature for 15 min. Additionally, experimentation determined the optimal siRNA concentration as 20 nM.

HSC3 and SCC25 cells in the logarithmic growth phase were plated into 6-well flat-bottom plates. At a density of 2 × 10^5^ cells/well, they were placed in 2 mL of antibiotic-free medium. Transfection complexes were introduced into the cells, which subsequently underwent culture at 37 °C in a humidified incubator with 5% CO_2_ for subsequent tests.

### 2.6. shRNA Transfection

The synthesized shRNA sequences (5′-CCGGCTGAGAGTATGCTTCAACAA CTCAGTTGTTGAGCATACTCTCAGCTTTT-3′) were inserted into a lentiviral vector (e.g., pLKO.1-puro). The constructed vector was then introduced into 293T cells. Lipofectamine 3000 (Thermo Fisher Scientific, Waltham, MA, USA; Cat. L3000-015) was employed to promote viral particle production. Following a 48-h incubation period, the culture supernatant was collected as well as subjected to centrifugation (1000× *g*, 10 min) for debris removal, resulting in purified lentiviral particles encapsulating the shRNA payload. To enhance transfection efficiency, target cells were transfected with the viral supernatant in the presence of Polybrene (2 μg/mL; Yeasen Biotechnology, Shanghai, China; Cat. No. 40804ES76). After transfection, the cells were subjected to a 7-day selection process using puromycin (Solarbio, Beijing, China, Cat. No. P8230-25mg; CAS No. 53-79-2), resulting in the generation of stable knockdown clones.

### 2.7. Functional Assays

Cell Proliferation: A CCK-8 assay kit (TargetMol, Wellesley Hills, MA, USA, Cat. CCK8-S016009110 mL) was employed to evaluate cell viability daily for 5 consecutive days, with OD values recorded at 450 nm.

Colony Formation: Six-well plates were injected with 1 × 10^3^ cells per well, and the cells were cultivated for 14 days. Colonies were then visually tallied, chemically fixed with 4% paraformaldehyde, as well as colored with 0.1% crystal violet (Beyotime; Shanghai, China, C0121).

Migration Assay: A sterile 200 µL pipette tip served to produce consistent wounds in confluent cell monolayers. A Leica DMi8 phase-contrast microscope captured wound closure images at 0 h and 24 h. Later, ImageJ 1.54g-based quantification methods were applied to calculate the cell migration rate.

Invasion Assay: 24-well plates (Corning, Cat. 3524) fitted with Transwell Permeable Supports (Falcon, Singapore; Cat. 353097) pre-coated with Matrigel (Corning; Cat. 354234) were utilized. Serum-free medium with 5 × 10^4^ cells/well was incorporated to the upper chamber and the lower one held 10% FBS as the chemoattractant. After 48 h, cells invading through the membrane were fixed using methanol, stained with 0.1% crystal violet, as well as counted under a Zeiss Axio microscope (Carl Zeiss AG, Jena, Germany)

### 2.8. Animal Model

Six-week-old NOD/SCID mice (6 mice per group) received submucosal tongue injections of shPLS3- or control-transduced SCC25 cells (1 × 10^6^ cells in 50 µL PBS). Meanwhile, six-week-old C57 mice (6 mice per group) were injected with stably transduced MOC1 mouse tongue squamous cell carcinoma cells (shNC or shPLS3) using the same method (1 × 10^6^ cells in 50 μL PBS). Starting on day 4, tumor volume in NOD/SCID mice was assessed every other day using the formula: V = (Length × Width*^2^*)/2. On day 12, the NOD/SCID mice were euthanized, and tumor tissues were collected for subsequent hematoxylin and eosin (H&E, Baton Rouge, LA, USA) staining and immunohistochemical (IHC) analysis. Similarly, tumors from C57 mice were measured for volume immediately after collection, and the tumor tissues were used for subsequent flow cytometry experiments.

### 2.9. Data Acquisition and Bioinformatics Analysis

This study systematically screened prognosis-associated genes in HNSCC through integrative multi-omics analyses. Core bioinformatics analyses leveraged RNA sequencing transcriptome profiles and clinical records of 502 HNSCC cases, which were sourced from The Cancer Genome Atlas repository (https://portal.gdc.cancer.gov/, accessed on 24 April 2024). Pan-cancer expression profiling of the target protein and survival association analyses were conducted via the TIMER2.0 platform (http://timer.cistrome.org/, accessed on 17 February 2025).

#### 2.9.1. Immune Microenvironment Profiling

A dual-algorithm strategy was employed to characterize the TIME:

CIBERSORT: Deconvolution analysis quantified the relative abundances for 22 immune cell types.

MCP-counter: Evaluated stromal/immune activity scores.

Data were normalized (Z-score) and correlated with target gene expression via Spearman’s rank correlation analysis.

#### 2.9.2. Regulatory Network and Co-Expression Analysis

The TISIDB platform (http://cis.hku.hk/TISIDB/, accessed on 25 July 2024) enabled the construction of interactive networks between the target protein and cytokine/chemokine receptor molecules.

LinkedOmics (https://linkedomics.org/, accessed on 24 April 2024) was applied to screen genes with differential co-expression (DEGs) between the PLS3-high as well as PLS3-low groups, based on criteria of FDR < 0.05 and |log2FC| > 1.

All analyses were conducted in the R 4.3.3 environment (https://www.r-project.org/, accessed on 4 February 2024). Tools employed involved packages such as limma, CIBERSORT, ggplot2,Seurat, andHarmony.

### 2.10. Statistical Analysis

Software called GraphPad Prism 10.0 was leveraged for statistical analyses. Two-tailed unpaired or paired Student’s *t*-tests were leveraged in two-group comparisons. Tukey’s post hoc test was leveraged following a one-way ANOVA for multiple-group analyses. The correlation between quantitative and categorical variables was evaluated using the Wilcoxon rank-sum test. Kaplan–Meier curves were drawn for survival analysis, log-rank testing was used to assess intergroup differences, and multivariate Cox proportional hazards regression was leveraged to further filter independent prognostic markers. Every in vitro experiment was carried out at least three times on its own. The data is displayed as Mean ± SD. The two-tailed criteria for statistical significance were * *p* < 0.05, ** *p* < 0.01, as well as *** *p* < 0.001.

## 3. Results

### 3.1. High PLS3 Expression Correlates with Poor Prognosis in HNSCC Patients

**Figure 1:**
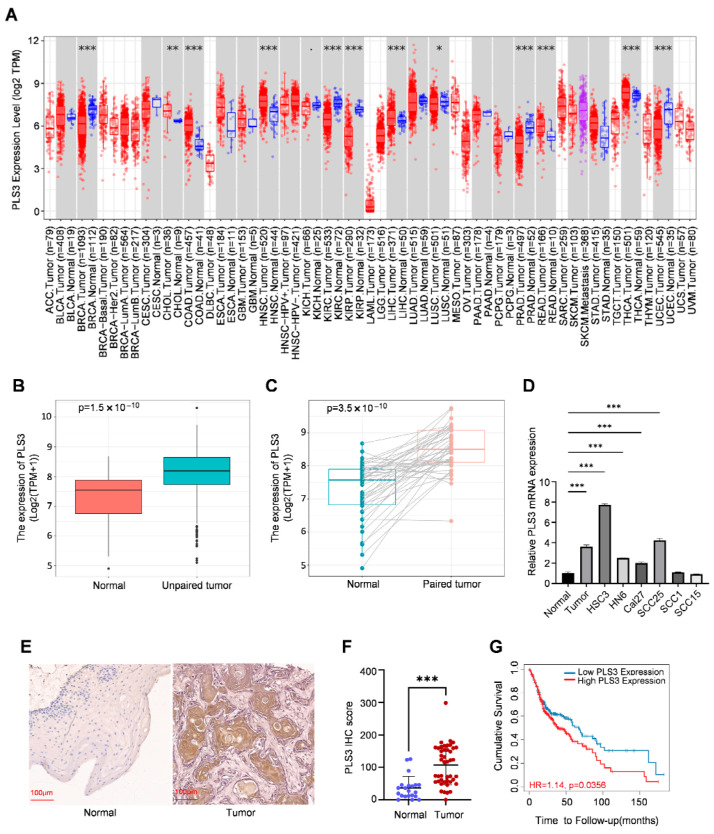
PLS3 is aberrantly expressed in HNSCC and associated with prognosis. (**A**) TIMER2.0 was utilized to detect the expression levels of PLS3 in different tumors within the TCGA database. (**B**,**C**) The expression level of PLS3 was analyzed in normal tissues and paired adjacent tumor tissues, including unmatched tissues (**B**) and matched tissues (**C**). (**D**) Relative PLS3 mRNA expression between normal tissue and HNSCC and cell lines. (**E**) Representative images of PLS3 expression in normal tissues and HNSCC tissues via IHC staining. Scale bar: 100 μm. (**F**) PLS3 IHC score assessment in normal and HNSCC tissues. (**G**) Survival analysis of patients with low vs. high PLS3 expression using Kaplan–Meier method. HR = hazard ratio. * *p* < 0.05, ** *p* < 0.01, *** *p* < 0.001. Data are presented as means ± SEM. Statistical analysis was performed via two-tailed Student’s *t*-test.

Interrogation of the TIMER2.0 database demonstrated that PLS3 is aberrantly overexpressed in various solid tumors when compared with normal tissues (Figure 1A). Notably, in HNSCC, PLS3 mRNA levels were notably higher in tumor tissues relative to adjacent normal controls (Figure 1B). Paired sample analysis further validated this tumor-specific upregulation (Figure 1C).

In a systematic exploration of PLS3 expression patterns in HNSCC, RT-qPCR analysis demonstrated PLS3 expression was remarkably elevated in tumor tissues and HNSCC cell lines (such as HSC3 and SCC25) when contrasted with normal epithelial tissues (Figure 1D). Furthermore, immunohistochemical (IHC) staining of 30 primary HNSCC tumor samples and 21 adjacent normal tissues demonstrated markedly elevated PLS3 protein expression in tumors. Semi-quantitative analysis using the H-score system (range: 0–300) confirmed significantly higher PLS3 levels in tumor tissues (Figure 1E,F). These results were highly consistent with transcriptomic data from the TCGA-HNSC cohort, providing multi-dimensional validation of aberrant PLS3 overexpression in HNSCC.

Clinical correlation analysis indicated high PLS3 expression closely related with advanced T stage (*p* = 0.033, Table 1). Survival analysis of the TCGA-HNSC cohort, stratified by median PLS3 expression, demonstrated that patients in the high-PLS3 group showed notably poorer overall survival than the low-PLS3 one (*p* < 0.01, log-rank test; Figure 1G). These findings collectively establish PLS3 as a novel prognostic biomarker in HNSCC, with its overexpression strongly linked to aggressive tumor progression and unfavorable clinical outcomes.

### 3.2. PLS3 Promotes Malignant Progression of HNSCC Through EMT

**Figure 2:**
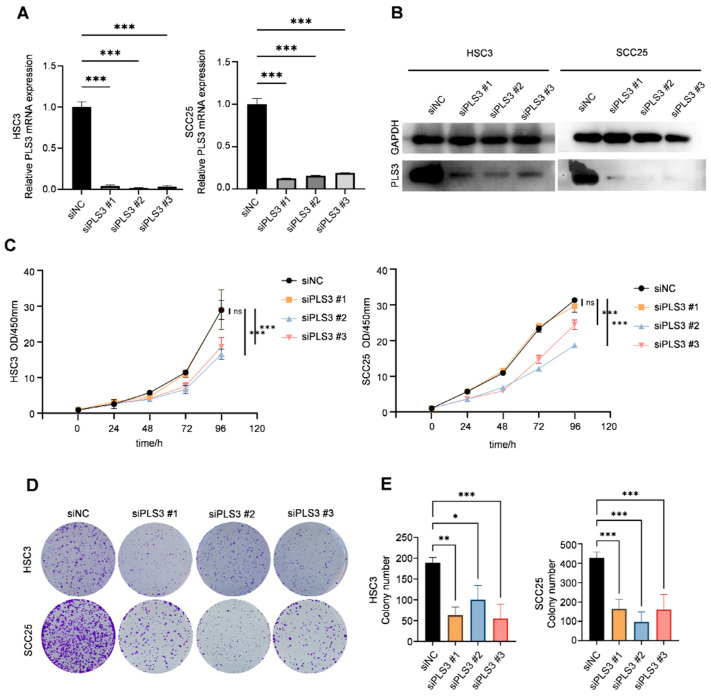
PLS3 knockdown impairs the proliferation and colony formation of HNSCC cells. (**A**) Relative PLS3 mRNA expression in HSC3 and SCC25 cells transfected with siPLS3 #1, siPLS3 #2, siPLS3 #3, or siNC (negative control siRNA), detected via qRT-PCR. (**B**) Western blot analysis of PLS3 protein expression in HSC3 and SCC25 cells after PLS3 knockdown. (**C**) CCK-8 assay evaluating cell proliferation of HSC3 and SCC25 cells with PLS3 knockdown at 0, 24, 48, 72, 96, and 120 h. (**D**) Colony formation images of HSC3 and SCC25 cells following PLS3 knockdown. (**E**) Statistical analysis of colony numbers in HSC3 and SCC25 cells with PLS3 knockdown. * *p* < 0.05, ** *p* < 0.01, *** *p* < 0.001. ns: no significance. Data are shown as means ± SEM, with statistical analysis performed via two-tailed Student’s *t*-test or ANOVA.

Previous studies have established PLS3 as a driver of aggressive progression in colorectal cancer, breast cancer, as well as other solid tumors. To investigate its functional role in HNSCC, we transiently transfected HSC3 and SCC25 cell lines with three specific siRNAs (siPLS3 #1–#3). RT-qPCR as well as Western blot validated the effective reduction in PLS3 mRNA (Figure 2A) as well as protein (Figure 2B) levels in all siRNA groups compared with controls.

**Figure 3:**
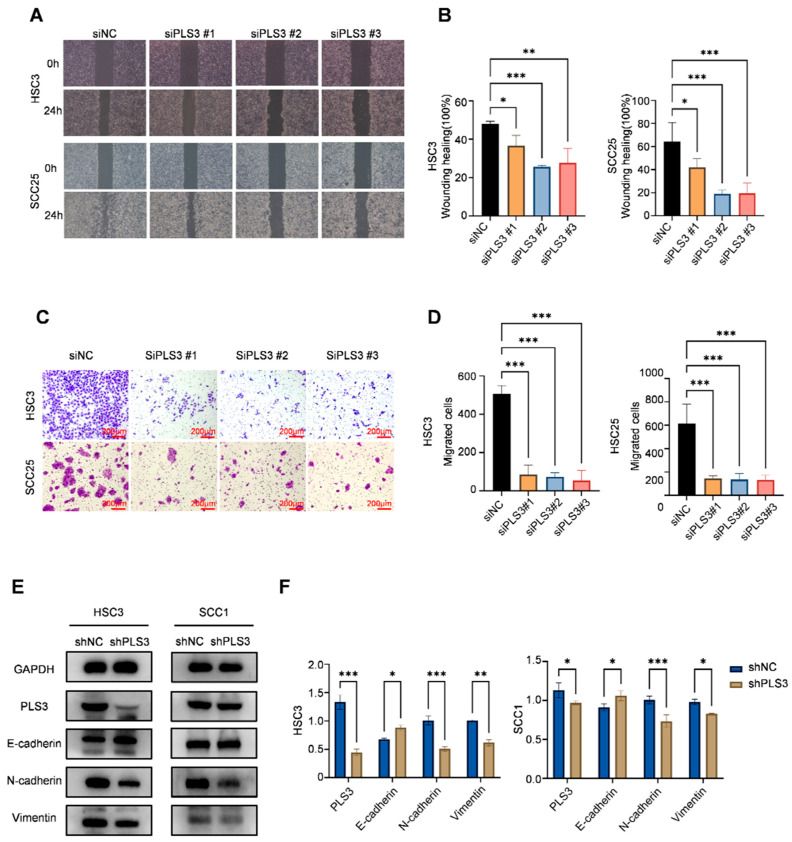
PLS3 knockdown inhibits migration, invasion, and epithelial–mesenchymal transition (EMT) of HNSCC cells. (**A**) Wound healing assay images of HSC3 and SCC25 cells at 0 h and 24 h after PLS3 knockdown (siPLS3 #1, siPLS3 #2, siPLS3 #3) or siNC treatment. (**B**) Statistical analysis of wound healing closure rates in HSC3 and SCC25 cells, showing reduced migration ability after PLS3 knockdown. (**C**) Transwell invasion assay images of HSC3 and SCC25 cells with PLS3 knockdown, indicating decreased invasive capacity. (**D**) Quantitative analysis of invaded cell numbers in HSC3 and SCC25 cells, verifying the inhibitory effect of PLS3 knockdown on invasion. (**E**) Western blot analysis of PLS3, E-cadherin, N-cadherin, and Vimentin expression in HSC3 and SCC1 cells after shPLS3 or shNC treatment. (**F**) Quantitative densitometric analysis of Western blot bands for EMT-related proteins in shNC and shPLS3 groups. * *p* < 0.05, ** *p* < 0.01, *** *p* < 0.001. Data are presented as means ± SEM, with statistical analysis via two-tailed Student’s *t*-test or ANOVA.

Functional cellular assays demonstrated that PLS3 knockdown significantly impaired key oncogenic behaviors in HNSCC cells. CCK-8 assays revealed that siPLS3 #2 and #3 significantly reduced cell proliferation over 96 h (Figure 2C). Colony formation assays demonstrated a marked decrease in clonogenic capacity upon PLS3 knockdown (Figure 2D,E). Wound healing assays showed attenuated scratch closure rates in siPLS3 groups at 24 h (Figure 3A,B). Transwell invasion assays further confirmed fewer cells penetrating the Matrigel-coated membranes after PLS3 silencing (Figure 3C,D).

Considering the critical function of EMT in tumor invasion and metastasis, we established stable PLS3-knockdown HSC3 and SCC25 cell models via lentiviral shRNA. Western blot results indicated that shPLS3 cells exhibited heightened expression of the epithelial marker E-cadherin, diminishing that of mesenchymal markers N-cadherin as well as Vimentin (Figure 3E,F). Notably, these findings present the first validation that PLS3 drives invasive traits in HNSCC by triggering EMT, thus unraveling a molecular mechanism behind its tumor-promoting function.

### 3.3. Knockdown of PLS3 Inhibits HNSCC Growth in Vivo

**Figure 4:**
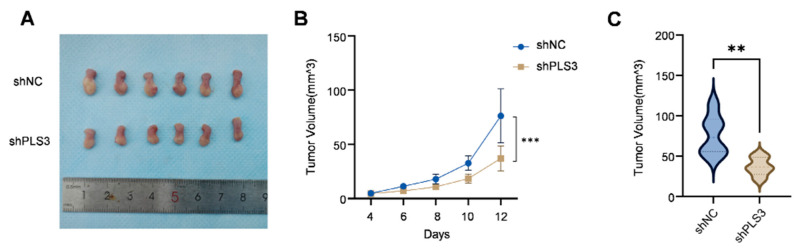

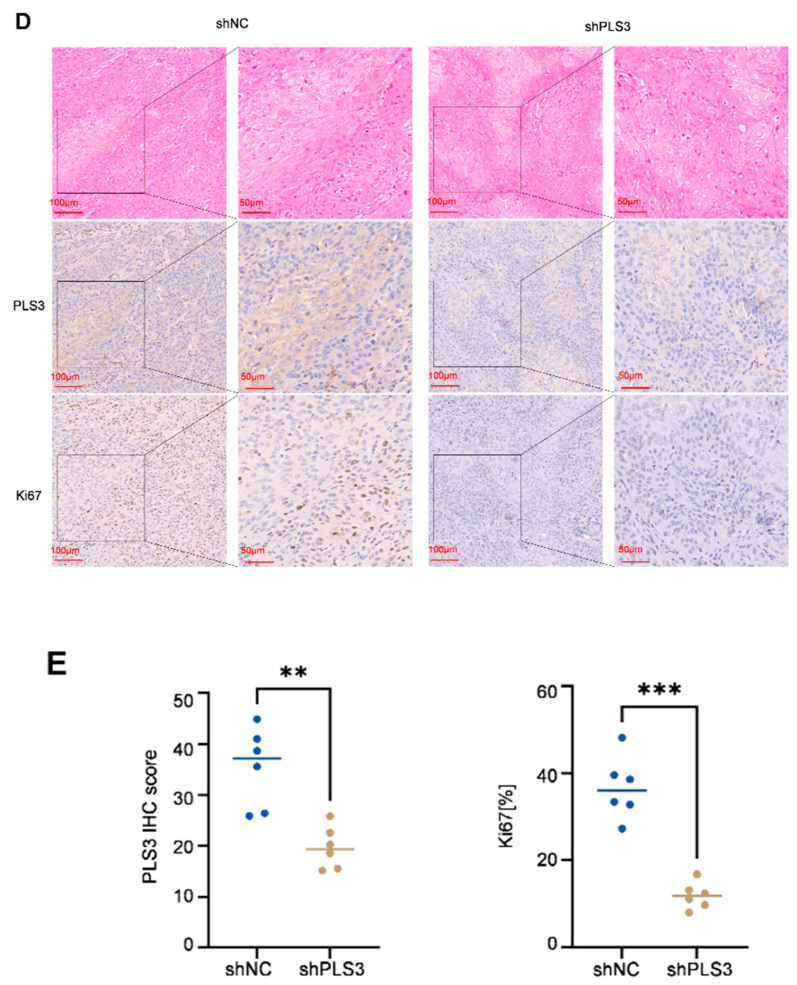
PLS3 knockdown inhibits tumor growth in the in vivo xenograft model. (**A**) Representative images of tumors from shNC (control) and shPLS3 (PLS3 knockdown) groups. (**B**) Tumor volume growth curves over time, comparing shNC and shPLS3 groups. (**C**) Statistical analysis of final tumor volumes, showing suppressed growth in the shPLS3 group. (**D**) HE staining and immunohistochemical staining for PLS3 and Ki67 in tumor tissues of shNC and shPLS3 groups, with scale bars indicating magnification. (**E**) Quantitative analysis of PLS3 IHC scores and Ki67-positive cell rates, revealing reduced PLS3 expression and proliferation (Ki67) in the shPLS3 group. ** *p* < 0.01, *** *p* < 0.001. Data are presented as means, with statistical analysis performed via two-tailed Student’s *t*-test.

To verify the tumor-promoting function of PLS3 in vivo, an orthotopic tongue xenograft model was constructed in NOD/SCID mice (6 mice per group) using SCC25 cells. The shPLS3 group exhibited slower tumor growth rates compared to controls (Figure 4A), with significantly reduced tumor volumes at the experimental endpoint (day 12, Figure 4B,C). IHC analysis further confirmed that shPLS3 tumors displayed lower PLS3 H-scores and decreased proportions of Ki67-positive cells (Figure 4D,E), indicative of attenuated proliferative activity. These in vivo findings conclusively demonstrate that PLS3 promotes malignant progression in HNSCC, reinforcing its potential as a therapeutic target.

### 3.4. PLS3 Is Closely Related to Immune Regulation

**Figure 5:**
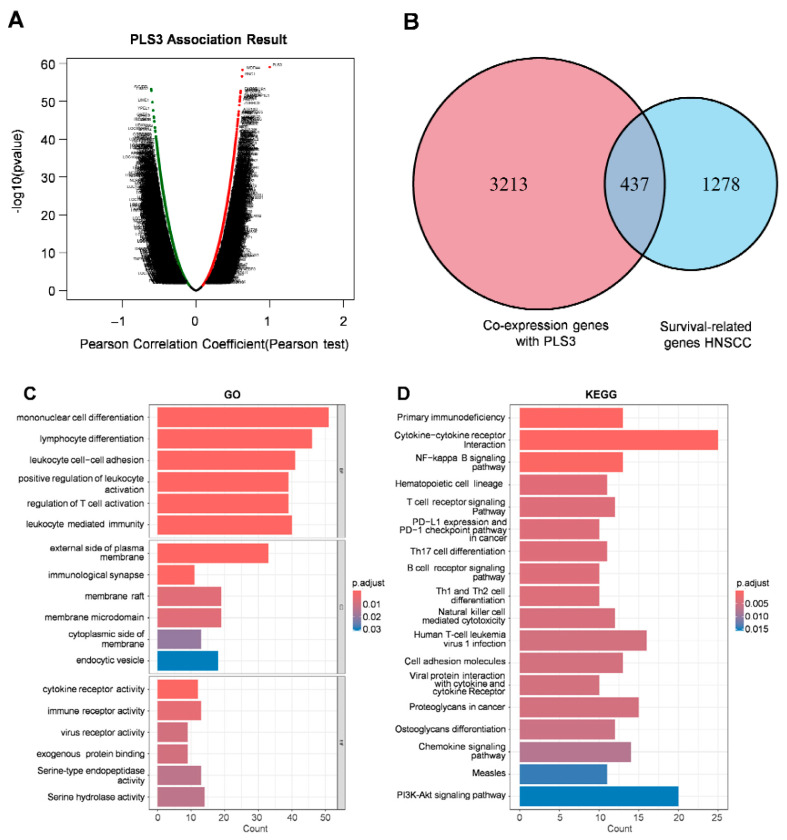

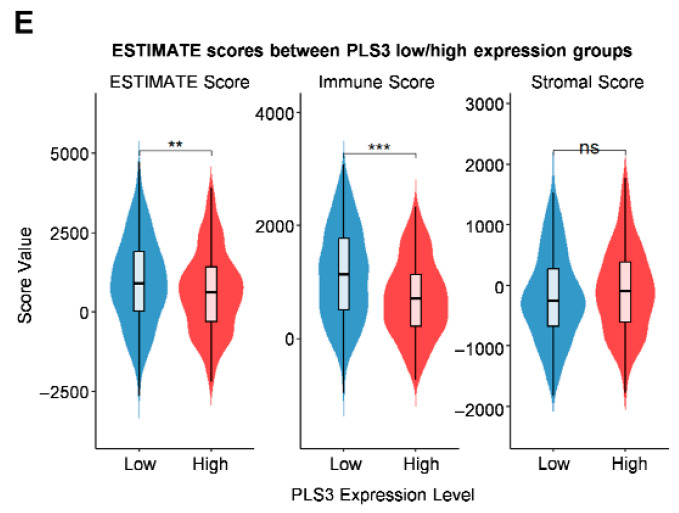
Functional enrichment analysis and immune correlation of PLS3 in HNSCC. (**A**) Scatter plot displaying the association results of PLS3, presenting Pearson correlation coefficient and −log10(*p*-value) via Pearson test. (**B**) Venn diagram showing the overlap between PLS3 co-expression genes and HNSCC survival-related genes. (**C**) GO (Gene Ontology) enrichment analysis of PLS3-associated genes, covering biological process, cellular component, and molecular function. (**D**) KEGG pathway enrichment analysis of PLS3-related genes, highlighting significantly enriched pathways. (**E**) Comparison of ESTIMATE scores (ESTIMATE Score, Immune Score, Stromal Score) between PLS3 low/high expression groups. In (**C**,**D**), *p*-adjust values reflect significance, with color gradients indicating different *p*-adjust ranges. ** *p* < 0.01, *** *p* < 0.001, ns: no significance.

To elucidate the molecular mechanisms by which PLS3 drives HNSCC progression, we employed a multi-omics integrative strategy. Through differential expression analysis (|log2FC| > 1, FDR < 0.05) of the TCGA-HNSC cohort via LinkedOmics, 3650 genes showing differential expression (DEGs) were screened out between the PLS3-high and PLS3-low groups (Figure 5A). Subsequent intersection with 1715 prognosis-associated genes yielded 437 hub genes (Figure 5B). GO enrichment analysis indicated remarkable enrichment of the hub genes in immune-related biological processes, encompassing regulation of T cell activation and lymphocyte differentiation (Figure 5C). KEGG pathway analysis further highlighted their involvement in T cell receptor signaling, cytokine-cytokine receptor interaction, as well as PD-L1/PD-1 checkpoint pathways (Figure 5D).

**Figure 6:**
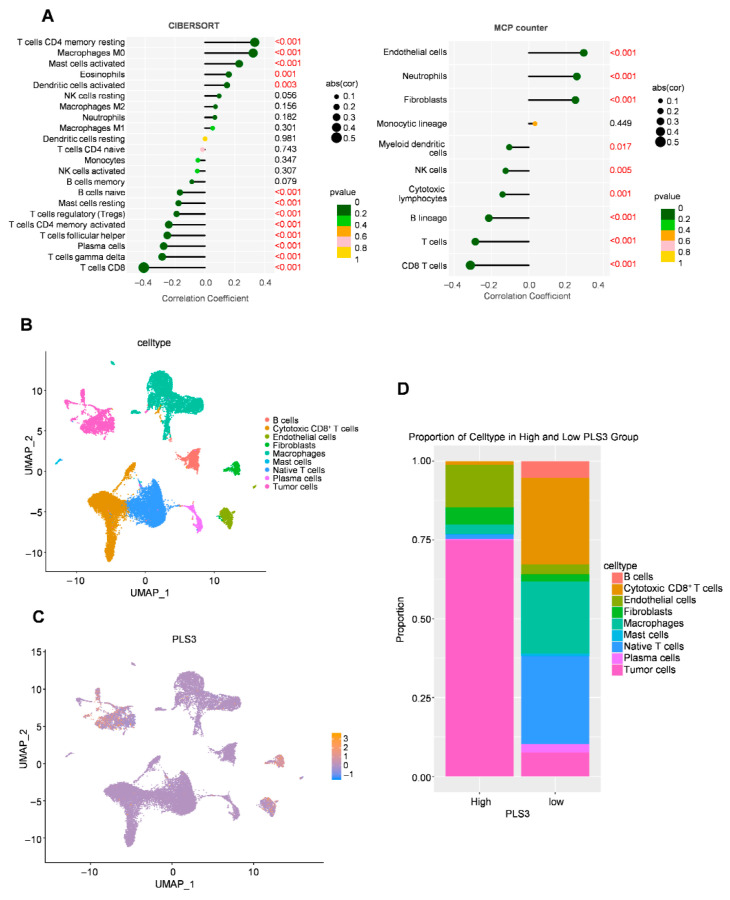
Immune cell infiltration analysis and cell interaction related to PLS3 in HNSCC. (**A**) Immune cell infiltration correlation analysis via CIBERSORT and MCP-counter, and red font indicates statistically significant values (*p* < 0.05). (**B**) UMAP (Uniform Manifold Approximation and Projection) visualization of cell types. (**C**) UMAP plot showing PLS3 expression distribution across cell clusters. (**D**) Proportion of cell types in high vs. low PLS3 expression groups, highlighting compositional differences.

Via the ESTIMATE algorithm, quantitative analysis of the tumor microenvironment showed, for the first time, that PLS3-high groups had significantly lower immune scores (*p* < 0.001) compared to controls (Figure 5E), indicating PLS3 might promote immune evasion by inhibiting anti-tumor immune cell infiltration. To explore the regulatory mechanisms of PLS3 on the immune microenvironment (IME), two algorithms: CIBERSORT and MCP-counter were used to assess immune cell infiltration patterns. Both analyses uncovered differing immune cell infiltration modes between PLS3-high and PLS3-low groups. Notably, the PLS3-high group displayed a remarkable inverse correlation with CD8^+^ T cell quantity (Figure 6A).

To delineate PLS3’s regulatory role in the TME at single-cell resolution, we performed UMAP-based dimensionality reduction and unsupervised clustering, resolving spatial distributions of key immune and stromal populations, encompassing cytotoxic CD8^+^ T, B cells, endothelial cells, as well as macrophages (Figure 6B). Single-cell transcriptional profiling identified heterogeneous PLS3 expression across cellular subsets, with predominant enrichment in tumor cells and fibroblasts clusters (Figure 6C). Cell type proportion analysis revealed that PLS3-high group exhibited elevated tumor cells proportions alongside depleted cytotoxic CD8^+^ T and NK cells compared to PLS3-low group (Figure 6D).

**Figure 7:**
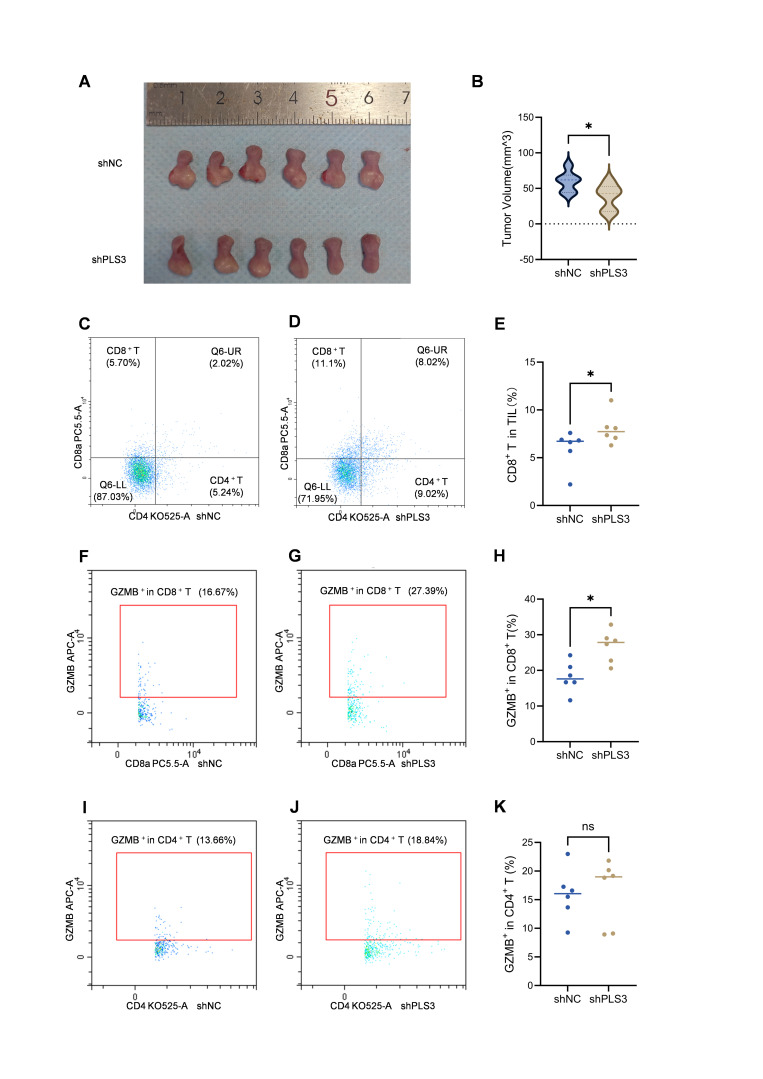
PLS3 knockdown inhibits tumor growth and enhances CD8^+^ T cell infiltration and cytotoxicity in xenograft models. (**A**) Representative images of tumors isolated from shNC and shPLS3 groups on day 12. (**B**) Quantitative analysis of tumor volume in shNC vs. shPLS3 groups (mean ± SD; * *p* < 0.05). (**C**,**D**) Flow cytometry plots showing the proportion of CD8^+^ T and CD4^+^ T cells in tumor-infiltrating lymphocytes (TILs) of shNC (**C**) and shPLS3 (**D**) groups. (**E**) Statistical comparison of CD8^+^ T cell proportion in TILs between shNC and shPLS3 groups (* *p* < 0.05). (**F**,**G**) Flow cytometry plots showing GZMB^+^ expression in CD8^+^ T cells of shNC (**F**) and shPLS3 (**G**) groups. (**H**) Statistical analysis of GZMB^+^ CD8^+^ T cell proportion in TILs between shNC and shPLS3 groups (* *p* < 0.05). (**I**,**J**) Flow cytometry plots showing GZMB^+^ expression in CD4^+^ T cells of shNC (**I**) and shPLS3 (**J**) groups. (**K**) Statistical comparison of GZMB^+^ CD4^+^ T cell proportion in TILs between shNC and shPLS3 groups (ns: no significance).

In the orthotopic tongue tumor model using C57 mice, results consistent with those in immunodeficient NOD/SCID mice were observed: tumor volume in the shPLS3 group was smaller than in the shNC group (Figure 7A,B). Flow cytometry analysis of tongue tumor tissues revealed a higher proportion of CD8^+^ T cells among tumor-infiltrating immune cells in the shPLS3 group (Figure 7C–E), while no significant difference was observed in CD4^+^ T cell proportion (Supplementary Figure S1A). Further analysis of Granzyme B (GZMB) expression showed higher levels in CD8^+^ T cells from the shPLS3 group (Figure 7F–H), but no significant difference in CD4^+^ T cells (Figure 7I–K). Flow cytometry of peripheral blood indicated that both CD4^+^ and CD8^+^ T cell proportions were higher in the shPLS3 group (Supplementary Figure S1B–G), although GZMB expression in these cells showed no significant difference between the two groups (Supplementary Figure S1H,I).

These findings establish PLS3 as a microenvironmental orchestrator that drives HNSCC progression by rewiring tumor–immune–stromal crosstalk to foster immune-evasive niches.

### 3.5. Correlation of PLS3 Expression with Immune Characteristics

**Figure 8:**
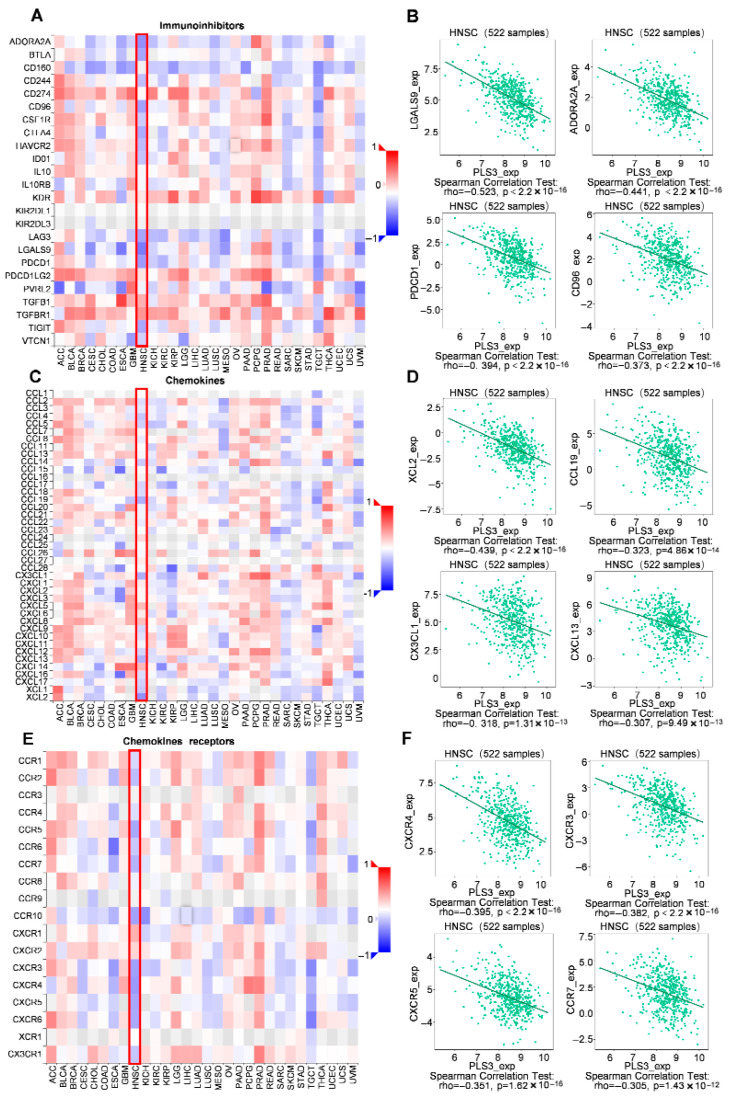
Correlation of PLS3 expression with immune characteristics. (**A**) Heatmap showing the correlation between PLS3 and immunoinhibitors across multiple tumor types, with HNSCC highlighted by a red box. (**B**) Scatter plots and Spearman correlation analysis of PLS3 expression with specific immunoinhibitors (LGALS9, ADORA2A, PDCD1, CD96) in 522 HNSCC samples. (**C**) Heatmap illustrating the correlation between PLS3 and chemokines, with HNSCC marked by a red box. (**D**) Scatter plots and Spearman correlation analysis of PLS3 expression with chemokines (XCL2, CCL19, CX3CL1, CXCL13) in HNSCC. (**E**) Heatmap depicting the correlation between PLS3 and chemokine receptors, with HNSCC highlighted via a red box. (**F**) Scatter plots and Spearman correlation analysis of PLS3 expression with chemokine receptors (CXCR4, CXCR3, CXCR5, CCR7) in HNSCC. In (**B**,**D**,**F**), rho represents the Spearman correlation coefficient, and *p*-value indicates statistical significance. The heatmaps (**A**,**C**,**E**) use color gradients (red to blue) to reflect positive to negative correlation strengths.

Given the key role of chemokine networks in regulating immune cell trafficking and tumor immune evasion [22,23], this study systematically delineated the relationship of PLS3 expression with immune phenotypes within the TME. Multidimensional analysis demonstrated that PLS3 expression exhibited significant negative correlations with key immune checkpoint molecules (*LGALS9*: ρ = −0.523, *p* < 2.2 × 10^−16^; *ADORA2A*: ρ = −0.441, *p* < 2.2 × 10^−16^; *PDCD1*: ρ = −0.394, *p* < 2.2 × 10^−16^; *CD96*: ρ = −0.373, *p* < 2.2 × 10^−16^) (Figure 8A,B), suggesting its potential to enhance antitumor immunity by suppressing immunosuppressive signaling. Mechanistic investigations further revealed that PLS3-high tumors displayed broad negative associations with chemokines (*XCL2*: ρ = −0.439, *p* < 2.2 × 10^−16^; *CCL19*: ρ = −0.323, *p* = 4.86 × 10^−14^; *CX3CL1*: ρ = −0.318, *p* = 1.31 × 10^−13^; *CXCL13*: ρ = −0.307, *p* = 9.49 × 10^−13^) and their receptors (*CXCR4*: ρ = −0.395, *p* = 2.2 × 10^−16^; *CXCR3*: ρ = −0.382, *p* < 2.2 × 10^−16^; *CXCR5*: ρ = −0.351, *p* = 1.62 × 10^−16^; *CCR7*: ρ = −0.305, *p* = 1.43 × 10^−12^) (Figure 8C–F). These findings uncover a dual cooperative mechanism by which PLS3 remodels the immune microenvironment: on the one hand, it downregulates immune checkpoint molecules to alleviate immunosuppression; on the other hand, it disrupts chemokine-receptor axes (e.g., CCL19-CCR7), thereby blocking effector immune cell recruitment and synergistically establishing an immune-privileged niche that fuels tumor progression.

## 4. Discussion

As a highly aggressive malignancy, HNSCC is typified by rapid progression and a complex immunosuppressive microenvironment that contributes significantly to its poor prognosis [24]. The identification of key molecular drivers and underlying mechanisms is crucial for improving treatment outcomes. PLS3, T-plastin, is an actin-binding protein belonging to the EF-hand domain with family. PLS3 resides in region 23 of the X chromosome’s long arm (Xq23) and encodes a protein crucial for the dynamic modulation of the cytoskeleton [15,25]. It has been reported that PLS3 affects cell motility, division, and maintenance of cell morphology by regulating the reorganisation of microfilaments in the cell [26,27]. Recently, PLS3’s role in tumors has increasingly drawn focus. Studies demonstrate PLS3 links to the development, metastasis, and poor prognosis of colorectal cancer [17,28], breast cancer [18], and pancreatic cancer [19]. However, its expression profile, functional significance, and therapeutic potential in HNSCC have remained poorly characterized until now. Therefore, we decided to perform a comprehensive and integrated bioinformatics analysis to study the biological roles as well as possible regulatory mechanisms of PLS3 in HNSCC.

Our study analyzed significant upregulation of PLS3 in HNSCC tumor tissues compared to normal ones. High PLS3 expression was strongly related with adverse clinical outcomes, encompassing advanced tumor stage as well as poor overall survival. The findings indicate PLS3 might act as a possible prognostic biomarker for HNSCC patients. The overexpression of PLS3 in HNSCC tissues may stem from multiple mechanisms, such as activating the Notch as well as PI3K/AKT signaling pathway. Prior research has suggested in thyroid cancer, PLS3 triggers the Notch signaling pathway to promote malignant progression [29]; in pancreatic cancer, it enhances cancer cell proliferation through PI3K/AKT signaling [19]. In accordance with the previous research, our present study has shown that PLS3 functions as a pro-oncogene in HNSCC. In vitro functional assays have demonstrated that PLS3 knockdown has a marked effect on the proliferation, cell migration, as well as invasion traits of HSC3 and SCC25 cell lines. These results have been corroborated in vivo using a murine orthotopic tongue xenograft model, where PLS3 knockdown has resulted in attenuated tumor growth.

At the molecular level, we found that PLS3 promotes HNSCC progression by activating the EMT program. EMT represents an active biological phenomenon where epithelial cells develop mesenchymal phenotypes. It enhances tumor cell invasive and migratory capabilities, remodels the immune microenvironment, and promotes therapy resistance, serving as a central mechanism driving tumor metastasis and malignant progression [30,31,32]. In the present study, decreasing PLS3 expression resulted in elevated levels of the epithelial marker E-cadherin as well as reduced ones of mesenchymal markers N-cadherin as well as Vimentin, complementing prior reports of PLS3 modulating EMT via the PI3K/AKT pathway. The results underscore the critical role of PLS3 in promoting HNSCC progression and indicate that targeting PLS3 could be a potential therapeutic approach for HNSCC.

Bioinformatics analyses further revealed that PLS3 overexpression is associated with an immunosuppressive microenvironment featuring reduced CD8^+^ T cell infiltration as well as suppressed chemokine expression. The immunosuppressive microenvironment is a hallmark of HNSCC, contributing to immune evasion and poor response to immunotherapy. Our findings suggest that PLS3 not only promotes tumor progression through EMT but also facilitates immune evasion by modulating the TME. This dual-axis mechanism provides a comprehensive understanding of how PLS3 drives HNSCC progression and emphasizes its possibility as a treatment target for both anti-tumor and immune-modulatory strategies.

Notably, the expression of PLS3 was particularly significant in immune cells, especially in T cells as well as macrophages [21,33]. This suggests PLS3 may have an important role in the immune response. Therefore, we assessed the connection between PLS3 expression and the immune microenvironment in HNSCC by means of dependent databases, including CIBERSORT, MCP-counter and TCGA. Bioinformatics analyses further revealed that high PLS3 expression was related with reduced CD8^+^ T cell infiltration as well as suppressed expression of key chemokines (e.g., CCL19) as well as their receptors (e.g., CCR7). KEGG enrichment analysis additionally demonstrated notable accumulation of PLS3-linked genes in cytokine-cytokine receptor interaction pathways and PD-L1/PD-1 immune checkpoint pathways. Flow cytometry analysis of our orthotopic tongue tumor model in C57 mice further confirmed that PLS3 knockdown positively correlated with increased CD8+ T cell infiltration in tumor tissues, along with elevated GZMB expression within these CD8+ T cells—a key cytolytic factor essential for their cytotoxic function. It is well known that immunosuppressive microenvironment is a hallmark of HNSCC, contributing to immune evasion and poor response to immunotherapy [34]. Over recent years, remarkable advancements have been attained in anti-tumor immunotherapy targeting HNSCC. PD-1/PD-L1 monoclonal antibodies, like pembrolizumab as well as nivolumab, which block immune checkpoint pathways, have become the treatment options for recurrent or metastatic HNSCC [6,35]. In the TME of HNSCC, CD8^+^ T cells serve as critical anti-tumor effector cells, mediating immune surveillance by recognizing and killing tumor cells. However, immunosuppressive cells like Tregs as well as TAMs, along with inhibitory molecules like PD-1/PD-L1, suppress CD8^+^ T cell activity, leading to tumor immune escape [36,37]. In our study, we found PLS3 is closely related with the TME, especially with the activation and infiltration of CD8^+^ T cells, suggesting that targeting PLS3 is likely to boost the anti-tumor activity of CD8+ T cells, thus improving the anti-tumor effectiveness in HNSCC. Subsequent animal experiments will be conducted to provide further validation of this finding.

Our findings suggest that PLS3 not only promotes tumor progression through EMT but also facilitates T cell anti-tumor immunity by modulating the tumor microenvironment. This dual-axis mechanism provides a comprehensive understanding of how PLS3 drives HNSCC progression and demonstrates its value as a therapeutic target for both anti-tumor as well as immune-modulatory strategies.

Limitations include the need to validate whether PLS3 directly regulates CCL19/CXCL1 transcription via chromatin immunoprecipitation (ChIP) or promoter-luciferase assays. Future studies will employ immunocompetent C57BL/6 mice with 4NQO-induced primary HNSCC models, combined with immune checkpoint inhibitors (e.g., PD-1/PD-L1 inhibitor Pembrolizumab [35], CTLA-4 inhibitor Ipilimumab [38]), to explore synergistic antitumor effects of PLS3 targeting and immunotherapy, providing preclinical evidence for clinical translation.

## 5. Conclusions

To conclude, this research demonstrates that PLS3 shows remarkable overexpression in HNSCC and maintains a close link with unfavorable clinical outcomes. PLS3 promotes HNSCC progression by activating the EMT program and creating an immunosuppressive microenvironment. Targeting PLS3 may deliver a promising method for improving HNSCC prognosis as well as treatment.

## Figures and Tables

**Table 1:** Correlation between PLS3 expression and the clinicopathological features of the HNSCC cases from TCGA.

Characteristic	Low Expression of PLS3	High Expression of PLS3	*p*-Value
n	237	237	
T Stage, n (%)			0.033
T1	20 (4.2%)	13 (2.7%)	
T2	78 (16.5%)	60 (12.7%)	
T3	66 (13.9%)	62 (13.1%)	
T4	73 (15.4%)	102 (21.5%)	
N Stage, n (%)			0.546
N0	122 (25.7%)	111 (23.4%)	
N1	36 (7.6%)	43 (9.1%)	
N2	76 (16%)	77 (16.2%)	
N3	3 (0.6%)	6 (1.3%)	
M Stage, n (%)			>0.999
M0	234 (49.4%)	235 (29.6%)	
M1	3 (0.6%)	2 (0.4%)	
Clinical Stage, n (%)			0.268
Stage I	10 (2.1%)	8 (1.7%)	
Stage II	52 (11%)	40 (8.4%)	
Stage III	52 (11%)	45 (9.5%)	
Stage IV	123 (25.9%)	144 (30.4%)	
Gender, n (%)			0.532
Female	59 (12.4%)	66 (13.9%)	
Male	178 (37.6%)	171 (36.1%)	
Smoker, n (%)			0.781
Yes	130 (27.4%)	134 (28.3%)	
No	107 (22.6%)	103 (21.7%)	
Alcohol History, n (%)			0.767
Yes	160 (33.8%)	164 (34.6%)	
No	77 (16.2%)	73 (15.4%)	
Age group, n (%)			0.52
>60	126 (26.6%)	119 (25.1%)	
≤60	111 (23.4%)	118 (24.9%)	
Age, median (IQR)	61 (54, 69)	61 (52,69)	0.444

## Data Availability

The original contributions presented in this study are included in the article/Supplementary Materials. Further inquiries can be directed to the corresponding authors.

## Supplementary Materials

The following supporting information can be downloaded at: https://www.mdpi.com/article/10.3390/cancers17233882/s1, Figure S1. Effects of PLS3 silencing on CD4^+^/CD8^+^ T cell subsets and GZMB expression in peripheral blood mononuclear cells (PBMCs).

## References

[1] Johnson D.E., Burtness B., Leemans C.R., Lui V.W.Y., Bauman J.E., Grandis J.R. (2020). Head and neck squamous cell carcinoma. Nat. Rev. Dis. Primers.

[2] Bray F., Laversanne M., Sung H., Ferlay J., Siegel R.L., Soerjomataram I., Jemal A. (2024). Global cancer statistics 2022: GLOBOCAN estimates of incidence and mortality worldwide for 36 cancers in 185 countries. CA A Cancer J. Clin..

[3] Chow L.Q.M. (2020). Head and neck cancer. N. Engl. J. Med..

[4] Cohen E.E.W., Bell R.B., Bifulco C.B., Burtness B., Gillison M.L., Harrington K.J., Le Q.-T., Lee N.Y., Leidner R., Lewis R.L. (2019). The society for immunotherapy of cancer consensus statement on immunotherapy for the treatment of squamous cell carcinoma of the head and neck (HNSCC). J. Immunother. Cancer.

[5] Cramer J.D., Burtness B., Le Q.T., Ferris R.L. (2019). The changing therapeutic landscape of head and neck cancer. Nat. Rev. Clin. Oncol..

[6] Ferris R.L., Blumenschein G., Fayette J., Guigay J., Colevas A.D., Licitra L., Harrington K., Kasper S., Vokes E.E., Even C. (2016). Nivolumab for recurrent squamous-cell carcinoma of the head and neck. N. Engl. J. Med..

[7] Litchfield K., Reading J.L., Puttick C., Thakkar K., Abbosh C., Bentham R., Watkins T.B.K., Rosenthal R., Biswas D., Rowan A. (2021). Meta-analysis of tumor- and T cell-intrinsic mechanisms of sensitization to checkpoint inhibition. Cell.

[8] Schreiber R.D., Old L.J., Smyth M.J. (2011). Cancer immunoediting: Integrating immunity’s roles in cancer suppression and promotion. Science.

[9] Puram S.V., Tirosh I., Parikh A.S., Patel A.P., Yizhak K., Gillespie S., Rodman C., Luo C.L., Mroz E.A., Emerick K.S. (2017). Single-cell transcriptomic analysis of primary and metastatic tumor ecosystems in head and neck cancer. Cell.

[10] Plaschka M., Benboubker V., Grimont M., Berthet J., Tonon L., Lopez J., Le-Bouar M., Balme B., Tondeur G., de la Fouchardière A. (2022). ZEB1 transcription factor promotes immune escape in melanoma. J. Immunother. Cancer.

[11] Mariathasan S., Turley S.J., Nickles D., Castiglioni A., Yuen K., Wang Y., Kadel E.E., Koeppen H., Astarita J.L., Cubas R. (2018). TGFβ attenuates tumour response to PD-L1 blockade by contributing to exclusion of T cells. Nature.

[12] Kumar V., Donthireddy L., Marvel D., Condamine T., Wang F., Lavilla-Alonso S., Hashimoto A., Vonteddu P., Behera R., Goins M.A. (2017). Cancer-associated fibroblasts neutralize the anti-tumor effect of CSF1 receptor blockade by inducing PMN-MDSC infiltration of tumors. Cancer Cell.

[13] Dongre A., Rashidian M., Reinhardt F., Bagnato A., Keckesova Z., Ploegh H.L., Weinberg R.A. (2017). Epithelial-to-mesenchymal transition contributes to immunosuppression in breast carcinomas. Cancer Res..

[14] Terry S., Savagner P., Ortiz-Cuaran S., Mahjoubi L., Saintigny P., Thiery J.-P., Chouaib S. (2017). New insights into the role of EMT in tumor immune escape. Mol. Oncol..

[15] Wolff L., Strathmann E.A., Müller I., Mählich D., Veltman C., Niehoff A., Wirth B. (2021). Plastin 3 in health and disease: A matter of balance. Cell. Mol. Life Sci..

[16] Yokobori T., Iinuma H., Shimamura T., Imoto S., Sugimachi K., Ishii H., Iwatsuki M., Ota D., Ohkuma M., Iwaya T. (2013). Plastin3 is a novel marker for circulating tumor cells undergoing the epithelial-mesenchymal transition and is associated with colorectal cancer prognosis. Cancer Res..

[17] Sugimachi K., Yokobori T., Iinuma H., Ueda M., Ueo H., Shinden Y., Eguchi H., Sudo T., Suzuki A., Maehara Y. (2014). Aberrant expression of plastin-3 via copy number gain induces the epithelial-mesenchymal transition in circulating colorectal cancer cells. Ann. Surg. Oncol..

[18] Ueo H., Sugimachi K., Gorges T.M., Bartkowiak K., Yokobori T., Müller V., Shinden Y., Ueda M., Ueo H., Mori M. (2015). Circulating tumour cell-derived plastin3 is a novel marker for predicting long-term prognosis in patients with breast cancer. Br. J. Cancer.

[19] Xin Z., Li D., Mao F., Du Y., Wang X., Xu P., Li Z., Qian J., Yao J. (2020). PLS3 predicts poor prognosis in pancreatic cancer and promotes cancer cell proliferation via PI3K/AKT signaling. J. Cell Physiol..

[20] Cui X., Zhang Y., Zhang L., Liu J., Bai Y., Cui Y., Wang B., Zhang S., Li X. (2023). Role of LONP2 in head and neck squamous cell carcinoma. Gene.

[21] Suma S., Suehara Y., Fujisawa M., Abe Y., Hattori K., Makishima K., Sakamoto T., Sawa A., Bando H., Kaji D. (2024). Tumor heterogeneity and immune-evasive T follicular cell lymphoma phenotypes at single-cell resolution. Leukemia.

[22] Ozga A.J., Chow M.T., Luster A.D. (2021). Chemokines and the immune response to cancer. Immunity.

[23] Saxena S., Singh R.K. (2021). Chemokines orchestrate tumor cells and the microenvironment to achieve metastatic heterogeneity. Cancer Metastasis Rev..

[24] Wang G., Zhang M., Cheng M., Wang X., Li K., Chen J., Chen Z., Chen S., Chen J., Xiong G. (2021). Tumor microenvironment in head and neck squamous cell carcinoma: Functions and regulatory mechanisms. Cancer Lett..

[25] Shinomiya H. (2012). Plastin family of actin-bundling proteins: Its functions in leukocytes, neurons, intestines, and cancer. Int. J. Cell Biol..

[26] Zhong W., Neugebauer J., Pathak J.L., Li X., Pals G., Zillikens M.C., Eekhoff E.M.W., Bravenboer N., Zhang Q., Hammerschmidt M. (2024). Functional insights in PLS3-mediated osteogenic regulation. Cells.

[27] Pollard T.D., Cooper J.A. (2009). Actin, a central player in cell shape and movement. Science.

[28] Ning Y., Gerger A., Zhang W., Hanna D.L., Yang D., Winder T., Wakatsuki T., Labonte M.J., Stintzing S., Volz N. (2014). Plastin Polymorphisms Predict Gender- and Stage-Specific Colon Cancer Recurrence after Adjuvant Chemotherapy. Mol. Cancer Ther..

[29] Wang D., Liu J., Chen Y., Jia L., Zhao K., He X. (2024). PLS3 promotes papillary thyroid carcinoma progression by activating the notch signaling pathway. Environ. Toxicol..

[30] Lamouille S., Xu J., Derynck R. (2014). Molecular mechanisms of epithelial-mesenchymal transition. Nat. Rev. Mol. Cell Biol..

[31] Erin N., Grahovac J., Brozovic A., Efferth T. (2020). Tumor microenvironment and epithelial mesenchymal transition as targets to overcome tumor multidrug resistance. Drug Resist. Updat..

[32] Taki M., Abiko K., Ukita M., Murakami R., Yamanoi K., Yamaguchi K., Hamanishi J., Baba T., Matsumura N., Mandai M. (2021). Tumor immune microenvironment during epithelial-mesenchymal transition. Clin. Cancer Res..

[33] Yuan Y., Wang P., Zhang H., Liu Y. (2024). Identification of M2 macrophage-related key genes in advanced atherosclerotic plaques by network-based analysis. J. Cardiovasc. Pharmacol..

[34] Canning M., Guo G., Yu M., Myint C., Groves M.W., Byrd J.K., Cui Y. (2019). Heterogeneity of the head and neck squamous cell carcinoma immune landscape and its impact on immunotherapy. Front. Cell Dev. Biol..

[35] Chen S., Yang Y., Wang R., Fang J. (2023). Neoadjuvant PD-1/PD-L1 inhibitors combined with chemotherapy had a higher ORR than mono-immunotherapy in untreated HNSCC: Meta-analysis. Oral Oncol..

[36] Elmusrati A., Wang J., Wang C.-Y. (2021). Tumor microenvironment and immune evasion in head and neck squamous cell carcinoma. Int. J. Oral. Sci..

[37] Jie H.-B., Schuler P.J., Lee S.C., Srivastava R.M., Argiris A., Ferrone S., Whiteside T.L., Ferris R.L. (2015). CTLA-4^+^ regulatory T cells increased in cetuximab-treated head and neck cancer patients suppress NK cell cytotoxicity and correlate with poor prognosis. Cancer Res..

[38] Rowshanravan B., Halliday N., Sansom D.M. (2018). CTLA-4: A moving target in immunotherapy. Blood.

