# Supplementary material for: PLS3 Is a Prognostic Biomarker and Correlates with Immune Infiltrates in Head and Neck Squamous Cell Carcinoma
Source: Cancers (Basel). 2025 Dec 4;17(23):3882. doi: 10.3390/cancers17233882 (PMC12691376; doi:10.3390/cancers17233882)
Supplement: Supplementary file 1 [file cancers-17-03882-s001.zip › cancers-3656820-supplementary.pdf]

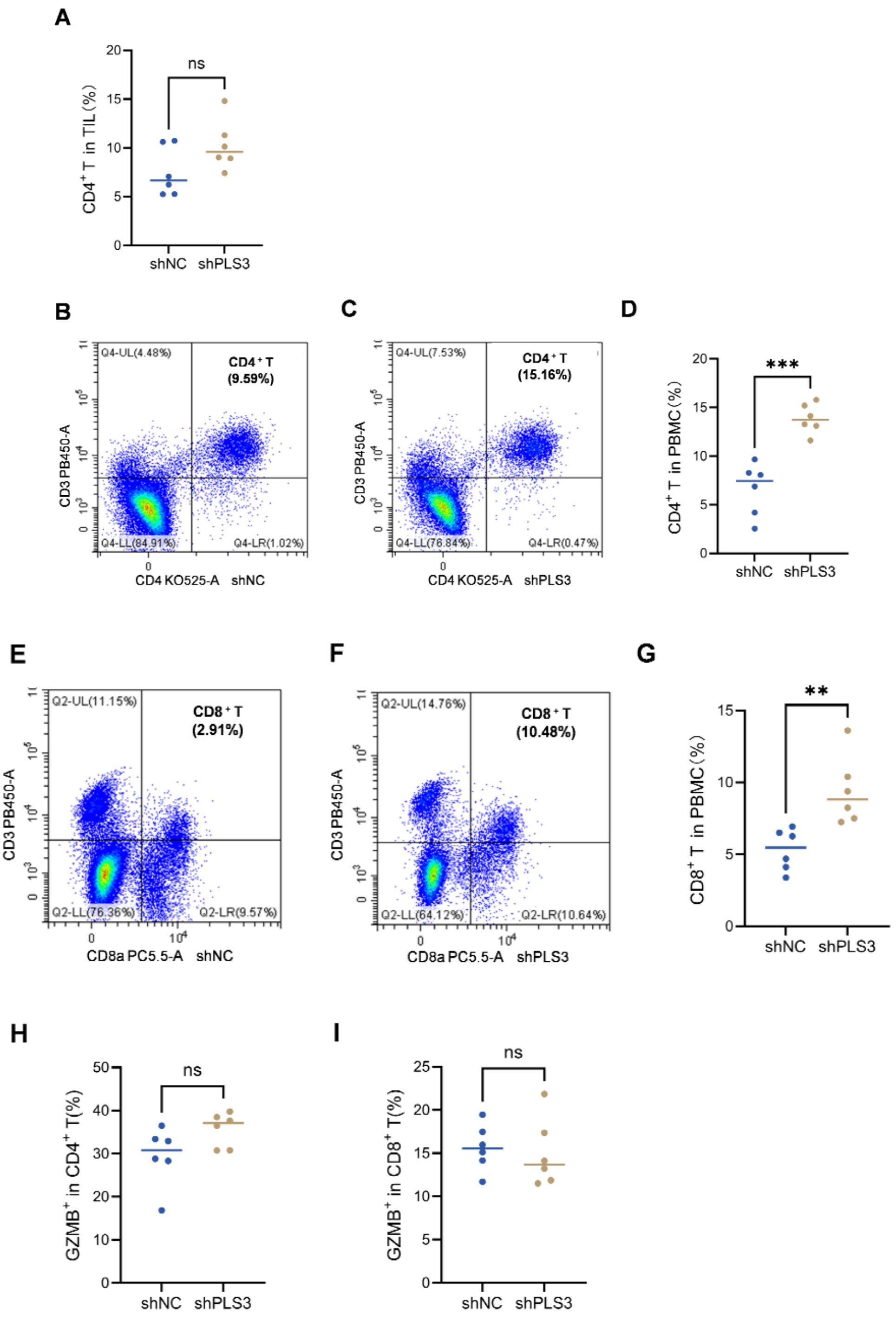

Figure S1. Effects of PLS3 silencing on CD4⁺/CD8⁺ T cell subsets and GZMB expression in peripheral blood mononuclear cells (PBMCs). (A). Statistical comparison of CD4⁺ T cell proportion in tumor-infiltrating lymphocytes (TILs) between shNC and shPLS3 groups (ns: no significance). (B,C). Flow cytometry plots showing the proportion of CD4⁺ T cell in PBMCs of shNC (B) and shPLS3 (C) groups. (D). Statistical analysis of CD4⁺ T cell proportion in PBMCs between shNC and shPLS3 groups (*** $p < 0.001$). (E, F). Flow cytometry plots showing the proportion of CD8⁺ T cell in PBMCs of shNC (E) and shPLS3 (F) groups. (G). Statistical comparison of CD8⁺ T cell proportion in PBMCs between shNC and shPLS3 groups (** $p < 0.01$). (H). Statistical analysis of GZMB⁺ CD4⁺ T cell proportion in PBMCs between shNC and shPLS3 groups (ns: no significance). (I). Statistical comparison of GZMB⁺ CD8⁺ T cell proportion in PBMCs between shNC and shPLS3 groups (ns: no significance).
